# Characterization of Interfacial Corrosion Behavior of Hybrid Laminate EN AW-6082 ∪ CFRP

**DOI:** 10.3390/ma17081907

**Published:** 2024-04-19

**Authors:** Alexander Delp, Shuang Wu, Jonathan Freund, Ronja Scholz, Miriam Löbbecke, Thomas Tröster, Jan Haubrich, Frank Walther

**Affiliations:** 1 Chair of Materials Test Engineering (WPT), TU Dortmund University, Baroper Str. 303, D-44227 Dortmund, Germany; 2 Automotive Lightweight Design (LiA), Paderborn University, Warburger Str. 100, D-33098 Paderborn, Germany; 3 German Aerospace Center (DLR e.V. Deutsches Zentrum für Luft-und Raumfahrt), Institute of Materials Research, Linder Höhe, D-51147 Cologne, Germany

**Keywords:** CFRP, corrosion exposure, EN AW-6082, galvanic corrosion, hybrid laminate, intrinsic bonding, laser structuring, linear sweep voltammetry, materials engineering, surface modification

## Abstract

The corrosion behavior of a hybrid laminate consisting of laser-structured aluminum EN AW-6082 ∪ carbon fiber-reinforced polymer was investigated. Specimens were corroded in aqueous NaCl electrolyte (0.1 mol/L) over a period of up to 31 days and characterized continuously by means of scanning electron and light microscopy, supplemented by energy dispersive X-ray spectroscopy. Comparative linear sweep voltammetry was employed on the first and seventh day of the corrosion experiment. The influence of different laser morphologies and production process parameters on corrosion behavior was compared. The corrosion reaction mainly arises from the aluminum component and shows distinct differences in long-term corrosion morphology between pure EN AW-6082 and the hybrid laminate. Compared to short-term investigations, a strong influence of galvanic corrosion on the interface is assumed. No distinct influences of different laser structuring and process parameters on the corrosion behavior were detected. Weight measurements suggest a continuous loss of mass attributed to the detachment of corrosion products.

## 1. Introduction

The improvement of lightweight structures for the automotive sector is an often-considered approach for reducing energy demand during operation. Sector-specific materials, e.g., carbon fiber-reinforced polymers (CFRP) with high specific strength, and light metals, such as aluminum with high impact strength and excellent formability, are predestined for synergetic hybridization. This combination of the individual advantages can be achieved by means of multi-material systems, which are also termed hybrid structures [1,2,3,4]. The joining technology is of utmost importance as it defines the load transmission within the hybrid structure. Common techniques are bolting, blind riveting, and welding, which lead to defects or thermal disruptions [5,6]. An alternative technique is adhesive bonding [7]. When adhesively bonded materials originating from different material groups, surface properties are important regarding joining technology, while different electrochemical potentials are challenging with respect to corrosion protection [8,9].

A commonly used aluminum alloy that inherits beneficial corrosion resistance and mechanical strength is EN AW-6082-T6 [10,11]. The surface functionalization of this alloy, which includes cleaning and surface structuring, is possible via laser pre-treatment [12,13]. Depending on the pretreatment parameters, this functionalization can increase the joint strength [14,15,16]. The combination of laser structuring of the metal component and co-curing of the metal sheet and CFRP allows a performant hybrid system made of EN AW-6082 ∪ CFRP [17]. Nevertheless, galvanic insulation of the carbon fibers and the aluminum alloy surface cannot be ensured. The enlarged surface roughness due to laser structuring enhances this effect by producing a larger surface area and material tips, which lead to a greater probability of direct fiber–metal contact. Therefore, it is necessary to characterize the corrosion behavior by means of short-term corrosion testing procedures, i.e., linear sweep voltammetry (lsv) [18,19], and long-term corrosion testing, i.e., corrosion exposure testing with a focus on laser structure. The corrosion reaction is triggered by the CFRP component, which itself usually remains undamaged, while the metal component dissolves [20]. EN AW-6082 is susceptible to pitting corrosion and fragmentation of the oxide layer in NaCl solutions [21]. The combination of both leads to enhanced corrosion processes [20]. This study aims to understand the short- and long-term corrosion morphology evolution of a hybrid laminate consisting of EN AW-6082 ∪ CFRP when considering different laser structuring parameters at the surface of the Al component. Excitation linear sweep voltammetry and corrosion exposure tests were performed. The mass changes were evaluated. The corrosion morphology evolution was characterized by means of light microscopy. The corrosion products were classified according to their appearance and composition via SEM and EDX.

## 2. Materials and Methods

### 2.1. Material

**Figure 1:**
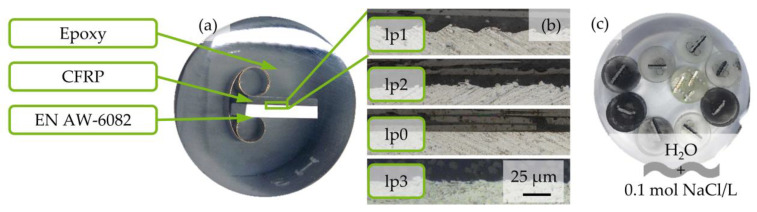
(**a**) EN AW-6082 ∪ CFRP embedded in epoxy resin and polished up to grain size of 1 mm, (**b**) micrograph of the three different laser parameter lp1–3 and as-rolled condition. CFRP component on top with fiber orientation horizontal and Al component (lp0) on the bottom, (**c**) experimental setup for corrosion exposure tests in aqueous solution of H_2_O and NaCl consisting of eight single epoxy-embedded specimens of EN AW-6082 ∪ CFRP and one epoxy-embedded specimen of three sheets of EN AW-6082.

The hybrid laminate consists of laser structured EN AW-6082 T6 sheet (Al), t = 2 mm, intrinsically bonded to five unidirectional layers CFRP Sigrapreg C (U230 0/NF E20/39%; SGL Carbon SE, Wiesbaden, Germany) in a P200S hot press (VOGT Labormaschinen GmbH, Berlin, Germany), as shown in Figure 1a. Laser structuring was realized with a Nd:YAG-Laser CL20 (Clean Lasersysteme GmbH, Herzogenrath, Germany), wavelength λ = 1064 nm, using three sets of parameters (lp), varying laser frequency f, laser power P, laser spot overlap o, and number of scans N, as listed in Table 1.

After laser structuring, CFRP was bonded to Al via co-curing. The hybrid systems, tested via corrosion exposure tests, were produced with a temperature T = 150 °C and pressure p = 0.5 MPa (for lp2) or p = 0.8 MPa (for lp1) for t = 5 min. Additional specimens with the following bonding parameters for lsv investigation of the influence of laser parameters were used: T = 160 °C, p = 0.8 MPa, t = 20 min. The resulting morphology of the laser structure can be seen in Figure 1b. Details of all used specimens are listed in Table 2, including the total exposure time Σt_exp_ and reference specimen.

### 2.2. Specimen Preparation

Eight specimens, four pretreated with lp1 and four pretreated with lp2, of EN AW-6082 ∪ CFRP laminate were prepared with two different fiber orientations, each (90° and 0° with regard to longitudinal fiber direction) for corrosion exposure testing (initial letter “K”). Two different fiber orientations were chosen to take the different distribution of carbon fiber volume content at the interface into account. For lsv, four specimens with lp0 to lp3 were prepared. Additionally, two EN AW-6082 specimens and one specimen of pure CFRP were provided. All specimens were embedded in epoxy resin EpoFix (Struers GmbH, Willich, Germany), ground, and polished up to particle size s = 1 μm. A prepared specimen is shown in Figure 1a. For characterization of the epoxy water absorption, a total of thirty cylindrical specimens of pure epoxy were prepared, using two diameters (d_1_ = 40 mm; d_2_ = 30 mm) and five filling levels (5/5 to 1/5), with a coverage of three specimens for each diameter. This resulted in six sets of specimens, three per diameter and five filling levels each, i.e., ten different masses. To ensure conductivity, the back side of specimens, used for lsv, were exposed by grinding and coated with conductive silver paint.

### 2.3. Experimental Setup

All weight measurements were performed using an analytical scale AUW220D (Shimadzu Corp., Kyoto, Japan), e = 1 mg; d = 0.01 mg. For iterative condition monitoring, a digital light microscope VHX-7000 (Keyence Corp., Osaka, Japan) was employed. Corrosion products were characterized via a scanning electron microscope (SEM) Mira 3 XMU (Tescan, Dortmund, Germany) and energy-dispersive X-ray spectroscopy (EDX) with Octane Elect Plus detector (Ametek GmbH, Meerbusch, Germany). Surface proportions of Al and CFRP were measured by means of ImageJ 1.53r.

For linear-sweep voltammetry, a three-electrode setup in a customized PMMA cell, described in [18], and a Gamry Interface 1000 potentiostat (Gamry Instruments Inc., Warminster, PA, USA) with Ag/AgCl as reference inside of a Luggin capillary (RE-1CP, ALS Co., Ltd., Tokyo, Japan), with a constant potential of 45.3 mV against saturated calomel electrode (RE-2BP, ALS Co., Ltd., Tokyo, Japan; +0.242 V vs. standard hydrogen electrode [23]) was employed.

### 2.4. Testing Method

Specimens for corrosion exposure testing were weighed, characterized via light microscopy, and placed in a beaker, as shown in Figure 1c, filled with 0.1 mol/L NaCl (purity > 99.8%, Ca < 0.01%, Mg < 0.002%, abherents < 0.0015%, batch 073196635; Carl Roth GmbH, Karlsruhe, Germany) in deionized H_2_O (conductivity κ < 2.5 μS/cm) equal to 11.688 g_NaCl_/2 L_H2O_. Conductivity of the mixed solution was measured (InLab 742 Mettler Toledo, DC, USA). All specimens were investigated via light microscopy after 24 h, 48 h, 72 h, 96 h, and 168 h. Additionally, four selected specimens were investigated after 336 h, 504 h, and 744 h to characterize the long-term corrosion evolution at the surface. Before light microscopy analysis, specimens were dipped into deionized H_2_O, air-dried, and weighed. All exposed specimens were replaced in the beaker after light microscopy measurements. The medium was replaced every 168 h. To prevent movement, evaporated water was not replaced. The short-term corrosion behavior of K1lp1 and K2lp2 (see Table 1) was investigated by means of lsv in accordance with ISO 17475 [24] before corrosion exposure tests and after t_exp_ = 168 h. Further specimens, no. lp0 to lp3, CFRP, and Al (see Table 1) were investigated by means of lsv. After a 1 h set-up time to reach open circuit potential (OCP), the measurement was realized with a potential feed ΔĖ = 1 mVs^−1^ and a potential range ΔE of ±300 mV vs. OCP. After the first lsv of K1lp1 and K2lp1, before corrosion exposure, the specimens were polished again. The second measurement was conducted on the corroded surface. Specimens lp0 to lp3 were tested three times each. After each lsv, the surface was polished again.

## 3. Results

### 3.1. Specimen Dimensions

The diameter and height of embedded specimens, as well as the area of the CFRP and Al fractions, were measured and are listed in Table 3. Due to the geometries of the initial EN AW-6082 ∪ CFRP with regard to fiber orientation, the volume of specimens with 90° orientation is higher, with areas of approximately 11–12 mm^2^ CFRP and 18–19 mm^2^ Al, while for 0° orientation, the areas are approximately 29–32 mm^2^ CFRP and 19–20 mm^2^.

### 3.2. Weight Measurement

**Figure 2:**
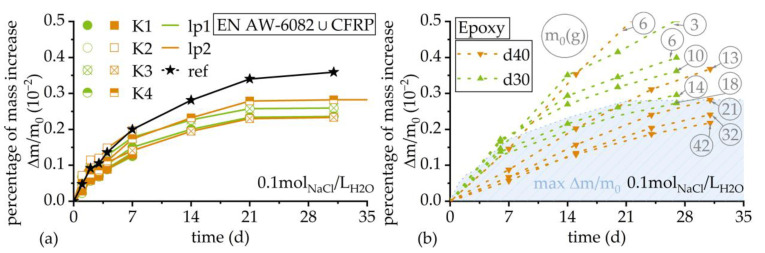
(**a**) Percentage increase in corrosion exposure specimen mass with regard to initial weight over time. The lines between measurements describe linear approximations for enhanced visualization. (**b**) Percentage increase in pure epoxy specimens with regard to initial weight over time. The initial weight m_0_ is listed in grey, inside a circle. The maximum percentage of mass increase in corrosion exposure specimens is plotted as blue area. Each triangle describes one measurement. The lines between triangles describe linear approximations for enhanced visualization. Two different diameters, d40 = 40 mm and d30 = 30 mm, were considered. The conductivity of the solution continuously increased, while the water volume decreased due to evaporation. This led to an increase in NaCl concentration from c_0_ = 0.10 mol_NaCl_/L_H2O_ to c_0_ = 0.11 mol_NaCl_/L_H2O_ within one week, which is shown in Table 5.

The weights of the specimens continuously increased in a range of 0.04 g during the testing period. Detailed records and are shown in Table 4 for over a period of 744 h, i.e., 4 weeks. The percentage increases were calculated with regard to initial weights of 0.25–0.27%, while the mass increase for reference EN AW-6082 reached Δm/m_0_ = 0.38%. This is shown in Figure 2a. The weight increase in reference specimens was distinctly higher than the weight increases in hybrid specimens. Investigations with pure epoxy indicate a continuous solution absorption during a time period of 31 days. The percentage increases were calculated with regard to initial weights for the arithmetic mean and fluctuated between Δm/m_0_ = 0.2% for the highest initial weight of approximately m = 42 g to Δm/m_0_ = 0.5% for an initial weight of m = 6 g, as shown in Figure 2b. The percentage weight increase is generally higher for specimens with lower initial weights. The blue area in Figure 2b represents the maximum percentage of weight increase, which was measured for hybrid specimens K4lp2 in corrosion exposure tests.

### 3.3. Linear Sweep Voltammetry

**Figure 3:**
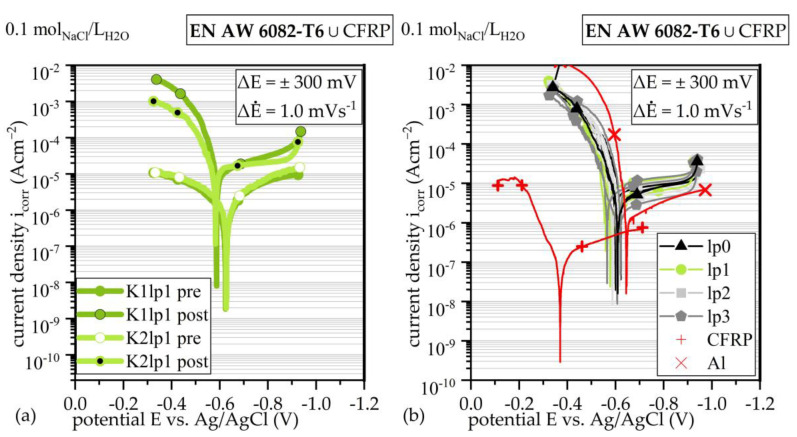
Tafel plots of lsv with logarithmic ordinate for comparison of short-term corrosion behavior: (**a**) before (pre) and after (post) corrosion exposure testing and (**b**) short-term corrosion behavior with regard to different lp, as well as pure CFRP and Al.

The OCP for both fiber orientation showed comparable OCP and current density i_corr_ before exposure testing. This can be seen in Figure 3a. After one week of exposure testing, both Tafel plots, i.e., i_corr_ and OCP, reached comparable trajectories.

Comparing lp, lsv detects no differences within a statistical scatter, as shown via Tafel plots in Figure 3b. Pure Al shows higher OCP, while i_corr_ was within a comparable range to the hybrid material. Lsv on CFRP shows distinct differences, as shown in [18]. When comparing the different production parameters between K-series and lp-series (compare Figure 3a,b), no remarkable differences were detectable. Due to the strong oscillation of the OCP in the presence of carbon fibers and the passivation effects of Al, a reliable determination of the corrosion current density, and therefore determination of the mass loss based on Faraday’s law, was not possible.

### 3.4. Microscopic Analyses

**Figure 4:**
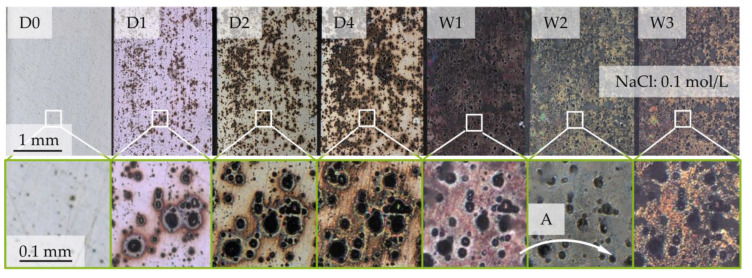
Depiction of corrosion evolution at EN AW-6082 over a time period of three weeks with recordings before exposure (D0), after one, two, and four days (D1, D2, D4) and after one, two, and three weeks (W1, W2, W3). Recorded with confocal light source. The evolution of one example area is marked via in a white box. (A) Traces the appearance of one pitting hole after two weeks via arrow.

The corrosion evolution during exposure testing showed the continuous progress of corrosion development on the surface morphology over the first seven days until week one (W1) for all specimens. Between W1 and week two (W2), and between W2 and week three (W3), there is no distinct change of surface pit morphology, but an increase of corrosion products at EN AW 6082 ∪ CFRP. The evolution of the surface morphology at the reference specimens, as shown in Figure 4, starts at random corrosion nuclei and forms randomly distributed corrosion pits over the whole surface after 24 h (D1). With further corrosion progress, the corrosion pit diameters increase until W1. White, salt-like corrosion products develop at the rims of the corrosion pits, until the entire surface is covered at W1; see Figure 4. In this state, few new corrosion pits emerge (Figure 4 (A)) and the development of new corrosion products and morphology at the surface stagnates.

**Figure 5:**
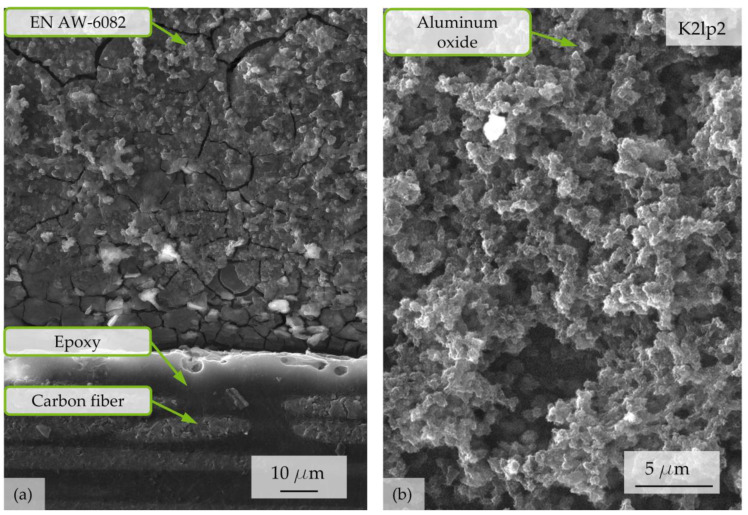
Representative SEM micrographs of surface morphology and corrosion products after an exposure time of t = 168 h on EN AW-6082 ∪ CFRP at specimen K2lp2. (**a**) Overview of the interface with visible trench at the Al component at the interface and corrosion products at the surface; (**b**) detailed overview over salt-like white corrosion products.

SEM investigations revealed that the surface structure of CFRP at the interface remains intact after a corrosion exposure time of t = 168 h, as shown in Figure 5. Despite no visible direct contact between carbon fibers and Al, the Al component is peeled off and forms a trench at the interface (see Figure 5a). The oxide layer shows cracks and aluminum oxides adhere randomly at the surface. Corrosion products, which appear white under light microscopy, are shown in Figure 5b and can be identified as aluminum oxides.

**Figure 6:**
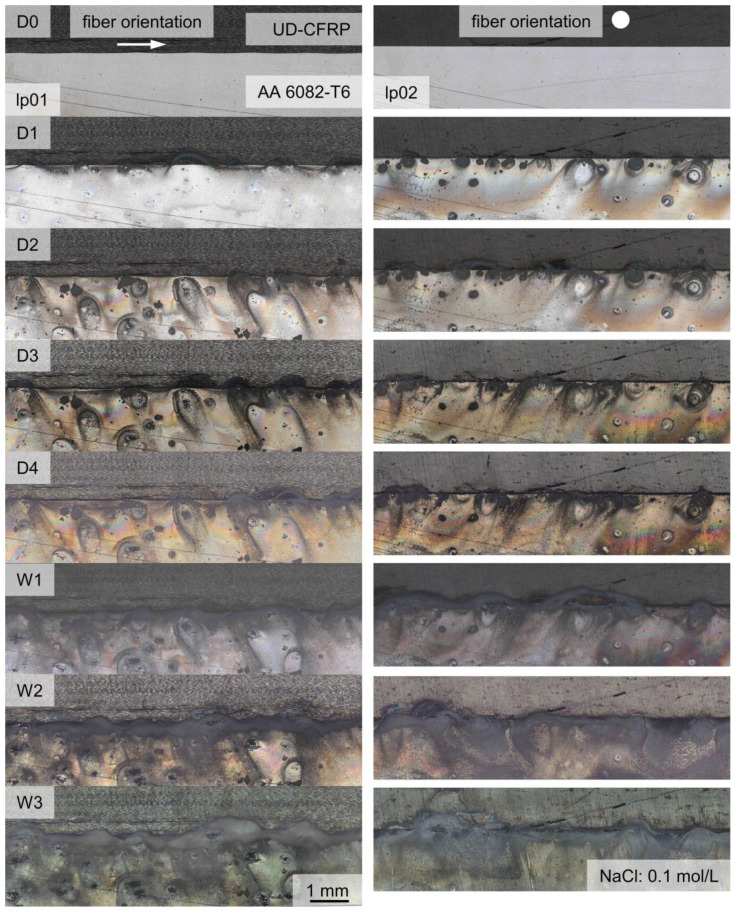
Depiction of corrosion evolution at EN AW-6082 ∪ CFRP over a time period of three weeks with recordings before exposure (D0), after one, two, three, and four days (D1, D2, D3, D4), and after one, two, and three weeks (W1, W2, W3) under consideration of fiber orientation and lp, using confocal microscopy for observation. The fiber orientation in length direction is marked with an arrow, the fiber orientation in view direction is marked via dot.

In the case of EN AW-6082 ∪ CFRP hybrids, the number of corrosion pits at the Al–overall surface is distinctly lower, but the diameter is higher. A representative overview for two different fiber orientations and lp is shown in Figure 6.

From D1, salt-like, white corrosion products develop around the interface, independent from fiber orientation or lp. The diameter of the appearing corrosion products is distinctly higher than the pitting hole itself. From D1 to D2, the quantity of detectable single corrosion pits next to the interface is higher when testing in the 0° fiber orientation. From D3, the distribution of corrosion products at the surface is similar, regardless of the lp and fiber orientations. From W1 to W3, the amount of corrosion products increases while the amount of corrosion pits remains similar. Only a minor growth in diameter, as visible in Figure 4, is observed. Due to the drying process (air-drying between the extraction actions), the corrosion products are U-shaped with streamlined distribution around the corrosion pits.

**Figure 7:**
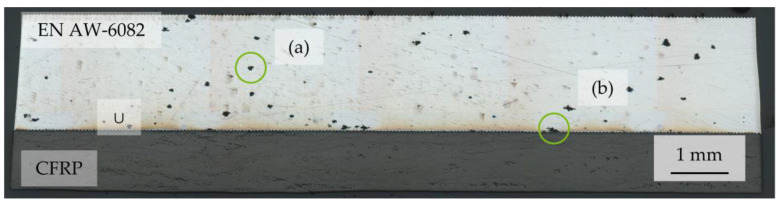
Example micrograph of EN AW-6082 ∪ CFRP after lsv measurement with (**a**) corrosion pits at the Al component, and (**b**) corrosion pits at the interface.

**Figure 8:**
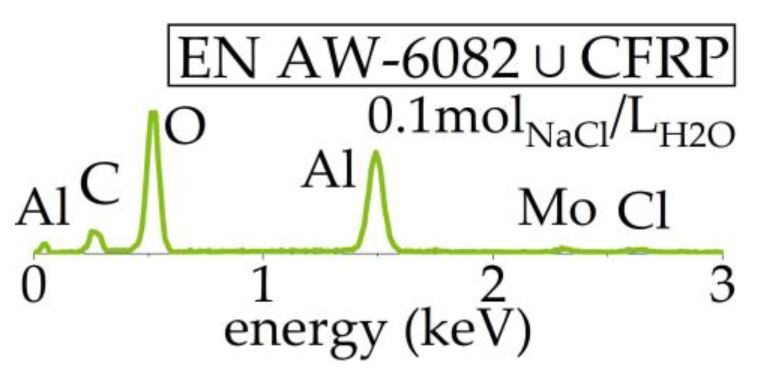
Representative results from EDX on corrosion products after corrosion exposure testing.

After lsv, Al exhibited no increased corrosion at the interface, as shown in Figure 7. Corrosion pits are distributed with a quantity comparable to corrosion exposure tests at EN AW-6082 ∪ CFRP, while no corrosion products adhere at the surface. Surfaces of pure Al show the same morphology as the surfaces of EN AW-6082 ∪ CFRP. EDX on corrosion products at the interface areas of EN AW-6082 ∪ CFRP revealed Al, C, and O, as well as small signals for Mo and Cl, but not Na, as shown in Figure 8.

## 4. Discussion

lsv and corrosion exposure testing did not show differences in the corrosion behavior for specimens with different laser pretreatments and production parameters. The ratio of the local surface increase at the interface to the total proportion of the Al component on the overall surface is small. Therefore, there is no quantifiable acceleration of corrosion processes during lsv and exposure testing with regard to different laser structuring after joining. Furthermore, it cannot be ensured that all embedded CFRP fibers are conductively connected, despite the conductive silver coating. Especially when orientated in a transverse direction, the inter-fiber contacts are responsible for electric conductivity. The investigations of Zappalorto et al. [25] and Zhao et al. [26] have proven the distinctly higher electric resistivity in out-of-plane orientation for CFRP. The reason for this is the local insulation of fibers against each other and against the Al component. When orientated in the transverse direction (the fiber’s longitudinal axis is transverse to specimen height), the fibers are not connected to the counter-side and are insulated against each other via the matrix. When orientated in the longitudinal direction (the fiber’s longitudinal axis is parallel to the specimen height), it is assumed that the higher conductivity of aluminum leads to the conduction of current via Al. As already stated in a previous publication [18], the OCP for EN AW-6082 ∪ CFRP is decreased compared to pure EN AW-6082, while the corrosion current density (*y*-axis shift, Figure 3) reaches a similar range. Comparable results for CFRP and EN AW-5754-O were observed by Li et al. [6] under the influence of a more aggressive medium (3.5 wt% NaCl). Additionally, it must be considered that it is not possible to distinguish between the conductive and non-conductive areas of the CFRP component after hybridization, i.e., the areas that were actually tested and those that remained non-conductively enclosed by the epoxy resin or are epoxy resin. It has to be assumed that the real surface area of EN AW 6082 ∪ CFRP exposed to corrosion processes and the medium was smaller than the total surface of EN AW-6082 ∪ CFRP, which was used to calculate i_cor_. Based on those considerations, it is assumed that the real corrosion current density has to be calculated on basis of the EN AW-6082 component of the EN AW-6082 ∪ CFRP hybrid. The narrowed surface area leads to a locally higher i_cor_ than assumed.

Weight measurements indicate a mass decrease by rinsing before microscopy. The epoxy resin of the mount absorbs more solution than the material of the embedded hybrid specimens’ increase in mass, indicating a more stable adhesion of corrosion products on EN AW 6082 compared to the surface of hybrid specimens. Therefore, continuous mass loss from EN AW-6082 ∪ CFRP is evident. This implies continuous corrosion processes at the aluminums surface at the interface.

The light microscopic analyses show a strong influence of the CFRP component on the corrosion evolution: large amounts of corrosion products continuously agglomerate at the interface, while the corrosion products at the reference material, pure Al, are evenly distributed. Additionally, only randomly distributed, individual points of the Al component with no direct contact to the interface develop corrosion pits. This is observed through lsv and corrosion exposure testing. SEM investigations confirm that the Al component dissolves while the CFRP component remains intact. The greatest loss of material occurs directly at the interface, which indicates a strong influence of galvanic corrosion. The lsv measurement of EN AW-6082 ∪ CFRP causes no agglomeration of corrosion products at the interface and generates a similar corrosion pit pattern to exposure testing. Therefore, it can again be assumed that the conductivity of the carbon fibers is restricted. Overall, there is a strong influence of galvanic corrosion. Due to the roughness peaks, the surface texture of EN AW-6082 provides randomly distributed contact points to carbon fibers (compare [5]). The corrosion morphology of the reference material during corrosion exposure testing, when compared to the morphology of EN AW-6082 ∪ CFRP, indicates that the interface acts like a sacrificial anode for the aluminum component without direct contact to CFRP at EN AW-6082 ∪ CFRP. Although the surface areas at the EN AW-6082 ∪ CFRP interface will have a different degree of direct connection to CFRP fibers, there is no global difference, as the PDP measurements have shown. Therefore, it can be assumed that the laser structuring has no effect on the global corrosion properties of the EN AW-6082 ∪ CFRP hybrid.

The EDX measurements suggest that the formed corrosion products consist of aluminum hydroxide. Traces of Cl further indicate the conversion of aluminum chlorohydrate [27]. Unlike in [6], without contact to galvanize steel, dawsonite could not be detected. The results also indicate that there are no remaining traces of NaCl after rinsing.

## 5. Conclusions

The results of this investigation of the corrosion evolution of a hybrid laminate consisting of laser-structured EN AW-6082 ∪ CFRP under the influence of NaCl electrolyte (0.1 mol/L) can be summarized as follows:

1. Galvanic coupling and passivation of the Al component, limited conductivity of the carbon fibers, and the random distribution of exposed fibers at specimen cut leads to high statistical scatter of the lsv measurement as well as uncertainties in the determination of the true surface and therefore limits the applicability of lsv for the hybrid material to qualitative comparisons.
2. A continuous mass loss was detected during corrosion exposure tests and could be allocated to the direct contact region of the interface, proving the dominance of galvanic corrosion on the long-term corrosion evolution of EN AW-6082 ∪ CFRP, while the corrosion mechanism of pure EN AW-6082 under same condition was identified as pitting corrosion. The interface acts comparably to a sacrificial anode for the Al base material.
3. Corrosion products were identified as aluminum oxides.

## Figures and Tables

**Table 1:** Parameter of laser structuring (lp) on EN AW-6082 T6 sheet consisting of laser frequency f, laser Power P, laser spot overlap o, and number of scans N [22].

Laser Parameter	f (kHz)	P (W)	o (10^−2^)	N
lp0	N/A	N/A	N/A	N/A
lp1	60	20	10	5
lp2	40	20	50	1
lp3	60	15	50	1

**Table 2:** Prepared specimen configuration and experimental procedure for corrosion exposure tests of EN AW-6082 ∪ CFRP in aqueous solution of 0.1 mol_NaCl_/L_H2O_, including an indication of whether lsv was performed.

Parameter Set	Fiber Direction (°)	Number	lsv	Total Exposure Time (h)
lp1	90	K1lp1	yes	168
lp1	0	K2lp1	yes	168
lp1	0	K3lp1	no	744
lp1	90	K4lp1	no	744
lp2	0	K1lp2	no	168
lp2	90	K2lp2	no	168
lp2	90	K3lp2	no	744
lp2	0	K4lp2	no	744
EN AW-6082	N/A	ref	no	744
lp0	0	lp0	yes	N/A
lp1	0	lp1	yes	N/A
lp2	0	lp2	yes	N/A
lp3	0	lp3	yes	N/A
EN AW-6082	N/A	Al	yes	N/A
CFRP	N/A	CFRP	yes	N/A

**Table 3:** Specimen dimensions and partial surfaces of CFRP and Al, as well as the total volume.

Number	Diameter (mm)	Height (mm)	Area CFRP (mm^2^)	Area Al (mm^2^)	Volume (mm^3^)
K1lp1	29.8	17.3	11.26	18.02	12,054
K2lp1	30.0	11.3	19.25	30.18	7988
K3lp1	29.8	12.5	19.70	31.11	8703
K4lp1	29.8	17.4	18.23	11.84	12,091
K1lp2	30.1	12.2	19.17	31.53	8660
K2lp2	29.8	17.1	10.47	18.64	11,935
K3lp2	30.0	17.1	10.94	18.58	12,080
K4lp2	29.9	12.7	17.82	29.63	8927
ref	30.0	11.4	00.00	00.00	8062

**Table 4:** Results of weight measurements during corrosion exposure testing.

Weight (g)	0 h (D0)	24 h (D1)	48 h (D2)	72 h (D3)	96 h (D4)	168 h (W1)	336 h (W2)	504 h (W3)	744 h (W4)
K1lp1	14.6750	14.6802	14.6844	14.6855	14.6880	14.6934	n.a.	n.a.	n.a.
K2lp1	10.5495	10.5519	10.5555	10.5567	10.5591	10.5637	n.a.	n.a.	n.a.
K3lp1	10.8513	10.8587	10.862	10.8629	10.8651	10.8706	10.8759	10.8792	10.8794
K4lp1	14.6666	14.6734	14.6788	14.6791	14.6817	14.6886	14.6959	14.7008	14.7012
K1lp2	10.5432	10.5462	10.5493	10.5505	10.5526	10.5568	n.a.	n.a.	n.a.
K2lp2	10.6009	10.6085	10.6130	10.6136	10.6166	10.6217	n.a.	n.a.	n.a.
K3lp2	13.6981	13.7044	13.709	13.7094	13.7129	13.7174	13.7248	13.7296	13.7301
K4lp2	11.1066	11.1121	11.1150	11.1162	11.1190	11.1258	11.1324	11.1376	11.1379
ref	10.1690	10.1740	10.1783	10.1797	10.1828	10.1893	10.1976	10.2035	10.2055

**Table 5:** Evaporated water over a time period of one week and the solution (s) conductivities κ at the start and end of solution usage with s1: t = 0 h…168 h; s2 t = 168 h…336 h; s3 t = 336 h…504 h, and s4 t = 504 h…744 h. Resulting c is calculated by c_0_ divided by remaining water volume.

Batch	Initial κ (mS/cm)	Final κ (mS/cm)	Remaining Water Volume (mL)	Resulting c (mol/L)
s1	10.50	11.24	1791	0.1117
s2	10.58	10.89	1817	0.1101
s3	10.29	11.76	1803	0.1109
s4	10.35	11.12	1763	0.1134

## Data Availability

The data presented in this study are available on request from the corresponding author. The data are not publicly available due to being part of an ongoing study.

## References

[1] Bader B., Türck E., Vietor T. (2019). Multi material design. A current overview of the used potential in automotive industries. Technologies for Economical and Functional Lightweight Design. Zukunftstechnologien für den Multifunktionalen Leichtbau.

[2] Min J., Li Y., Li J., Carlson B.E., Lin J. (2015). Friction stir blind riveting of carbon fiber reinforced polymer composite and aluminum alloy sheets. Int. J. Adv. Manuf. Technol..

[3] Vermeeren C.A.J.R. (2003). An historic overview of the development of fibre metal laminates. Appl. Compos. Mater..

[4] Malekinejad H., Carbas R.J.C., Akhavan-Safar A., Marques E.A.S., Castro Sousa F., da Silva L.F.M. (2023). Enhancing fatigue life and strength of adhesively bonded composite joints: A comprehensive review. Materials.

[5] Pramanik A., Basak A.K., Dong Y., Sarker P.K., Uddin M.S., Littlefair G., Dixit A.R., Chattopadhyaya S. (2017). Jointing of carbon fibre reinforced polymer (CFRP) composites and aluminium alloys—A review. Compos. Part A Appl. Sci. Manuf..

[6] Li S., Khan H.A., Hihara L.H., Cong H., Li J. (2018). Corrosion behavior of friction stir blind riveted AL/CFRP and Mg/CFRP joints exposed to a marine environment. Corros. Sci..

[7] Park S.Y., Choi W.J., Choi H.S., Kwon H., Kim S.H. (2010). Recent trends in surface treatment technologies for airframe adhesive bonding processing: A review (1995–2008). J. Adhes..

[8] Kwon D.S., Yoon S.H., Hwang H.Y. (2019). Effects of residual oils on the adhesion characteristics of metal-CFRP adhesive joints. Compos. Struct..

[9] Ireland R., Arronche L., Saponara V.L. (2012). Electrochemical investigation of galvanic corrosion between aluminum 7075 and glass fibre/epoxy composites modified with carbon nanotubes. Compos. Part B Eng..

[10] Xhanari K., Finšgar M. (2019). The corrosion inhibition of AA6082 aluminium alloy by certain azoles in chloride solution: Electrochemistry and surface analysis. Coatings.

[11] Lumley R. (2018). Fundamentals of Aluminium Metallurgy.

[12] Rechner R., Jansen I., Beyer E. (2010). Influence on the strength and aging resistance of aluminium joints by laser pre-treatment and surface modification. Int. J. Adhes..

[13] Heckert A., Zaeh M.F. (2014). Laser surface pre-treatment of aluminium for hybrid joints with glass fibre reinforced thermoplastics. Phys. Procedia.

[14] Steinert P., Dittes A., Schimmelpfennig R., Scharf I., Lampke T., Schubert A. (2018). Design of high strength polymer metal interfaces by laser microstructured surfaces. IOP Conf. Ser. Mater. Sci. Eng..

[15] Zhang Z., Shan J., Tan X., Zhang J. (2017). Improvement of the laser joining of CFRP and aluminum via laser pre-treatment. Int. J. Adv. Manuf. Technol..

[16] Schanz J., Meinhard D., Dostal I., Riegel H., De Silva A.K.M., Harrison D.K., Knoblauch V. (2022). Comprehensive study on the influence of different pretreatment methods and structural adhesives on the shear strength of hybrid CFRP/aluminum joints. J. Adhes..

[17] Wu S., Delp A., Freund J., Walther F., Haubrich J., Löbbecke M., Tröster T. (2023). Adhesion properties of the hybrid system made of laser-structured aluminium EN AW 6082 and CFRP by co-bonding-pressing. J. Adhes..

[18] Delp A., Freund J., Wu S., Scholz R., Löbbecke M., Haubrich J., Tröster T., Walther F. (2022). Influence of laser-generated surface micro-structuring on the intrinsically bonded hybrid system CFRP-EN AW 6082-T6 on its corrosion properties. Compos Struct..

[19] Narsimhachary D., Rai P.K., Shariff S.M., Padmanabham G., Mondal K., Basu A. (2019). Corrosion behavior of laser-brazed surface made by joining of AA6082 and galvanized steel. J. Mater. Eng. Perform..

[20] Song G.-L., Zhang C., Chen X., Zheng D. (2021). Galvanic activity of carbon fiber reinforced polymers and electrochemical behavior of carbon fiber. Corros. Commun..

[21] Li Z., Peng M., Wei H., Zhang W., Lv Q., Zhang F., Shan Q. (2023). First-principles study on surface corrosion of 6082 aluminum alloy in H+ and Cl medium. J. Mol. Struct..

[22] Freund J., Lützenkirchen I., Löbbecke M., Delp A., Walther F., Wu S., Tröster T., Haubrich J. (2023). Transferability of the structure-property relationships from laser-pretreated metal-polymer joints to aluminum-CFRP hybrid joints. J. Compos. Sci..

[23] McCafferty E. (2010). Introduction to Corrosion Science.

[24] (2006). German version EN ISO 17475:2008: Corrosion of Metals and Alloys—Electrochemical Test Methods—Guidelines for Conducting Potentiostatic and Potentiodynamic Polarization Measurements.

[25] Zappalorto M., Panozzo F., Carraro P.A., Quaresimin M. (2017). Electrical response of laminate with a delamination: Modelling and experiments. Compos. Sci. Technol..

[26] Zhang D.-Q., Li J., Joo H.G., Lee K.Y. (2009). Corrosion properties of Nd:YAG laser-GMA hybrid welded AA6061 Al alloy and its microstructure. Corros. Sci..

[27] Zhao Q., Zhang K., Zhu S., Xu H., Cao D., Zhao L., Zhang R., Yin W. (2019). Review on the electrical resistance/conductivity of carbon fiber reinforced polymer. Appl. Sci..

